# Oxidative Stress in the Male Germline: A Review of Novel Strategies to Reduce 4-Hydroxynonenal Production

**DOI:** 10.3390/antiox7100132

**Published:** 2018-10-03

**Authors:** Jessica L. H. Walters, Geoffry N. De Iuliis, Brett Nixon, Elizabeth G. Bromfield

**Affiliations:** Priority Research Centre for Reproductive Science, School of Environmental and Life Sciences, Discipline of Biological Sciences, University of Newcastle, Callaghan, NSW 2380, Australia; jwalters1@uon.edu.au (J.L.H.W.); geoffry.deiuliis@newcastle.edu.au (G.N.D.I.); Brett.nixon@newcastle.edu.au (B.N.)

**Keywords:** male fertility, oxidative stress, 4-hydroxynonenal (4HNE), arachidonate 15-lipoxygenase (ALOX15), lipid peroxidation, reactive oxygen species (ROS)

## Abstract

Germline oxidative stress is intimately linked to several reproductive pathologies including a failure of sperm-egg recognition. The lipid aldehyde 4-hydroxynonenal (4HNE) is particularly damaging to the process of sperm-egg recognition as it compromises the function and the stability of several germline proteins. Considering mature spermatozoa do not have the capacity for de novo protein translation, 4HNE modification of proteins in the mature gametes has uniquely severe consequences for protein homeostasis, cell function and cell survival. In somatic cells, 4HNE overproduction has been attributed to the action of lipoxygenase enzymes that facilitate the oxygenation and degradation of ω-6 polyunsaturated fatty acids (PUFAs). Accordingly, the arachidonate 15-lipoxygenase (ALOX15) enzyme has been intrinsically linked with 4HNE production, and resultant pathophysiology in various complex conditions such as coronary artery disease and multiple sclerosis. While ALOX15 has not been well characterized in germ cells, we postulate that ALOX15 inhibition may pose a new strategy to prevent 4HNE-induced protein modifications in the male germline. In this light, this review focuses on (i) 4HNE-induced protein damage in the male germline and its implications for fertility; and (ii) new methods for the prevention of lipid peroxidation in germ cells.

## 1. Introduction: Fertility and Oxidative Stress

A decline in fertility rates is becoming an increasingly prevalent issue worldwide, with current estimates indicating that 1 in every 6 couples experience issues with conception [1]. Furthermore, the contribution of male factor infertility accounts for up to half of these cases [2]. The leading cause of male infertility stems from a loss of sperm function, ultimately resulting in a loss of fertilization potential [3]. This loss in function is causatively linked to oxidative stress within the cell [4,5] driven by the presence and/or overproduction of intracellular reactive oxygen species (ROS). Reactive oxygen species are oxygen-containing molecules that can contain unpaired electrons (radicals) or be non-radical oxidizing agents [6]. The consequences of ROS are realized through redox reactions with a great number of biological substrates, producing either further reactive products or oxidized biomolecules. Within spermatozoa, low levels of ROS are essential for promoting key stages of development. For instance, ROS actively participate in metabolic pathways during sperm activation, which leads to cholesterol efflux, cyclic adenosine monophosphate (cAMP) production and tyrosine phosphorylation, important events that contribute to fertilization competence [5,7,8,9]. However, if intracellular ROS production escalates beyond the buffering antioxidant capacity of the cell in a state of oxidative stress, the redox biochemistry leads to damaging effects such as lipid peroxidation, organelle degradation, DNA damage and eventually cell death [10,11]. Typically, antioxidants, which counteract and protect against oxidative stress, are housed within the cytoplasm and mitochondria of somatic cells [12,13]. However, spermiogenesis, a process that gives rise to the unique architecture of mature spermatozoa, results in significant cytoplasmic depletion [14,15], thereby diminishing antioxidant capacity in the spermatozoon [16]. Furthermore, during testicular maturation, there is an enrichment of long chain poly-unsaturated fatty acids (PUFAs) in the sperm plasma membrane, which can serve as important substrates for lipid peroxidation [10]. Indeed, PUFAs such as arachidonic acid, linoleic acid and docosahexaenoic acid are enriched within the sperm plasma membrane [17,18], and can be broken down into cytotoxic lipid aldehydes that promote cellular damage and the dysregulation of cell function [19]. Common metabolites of lipid peroxidation within spermatozoa include reactive aldehyde compounds such as 4-hydroxynonenal (4HNE) and malondialdehyde (MDA) [19,20,21]. Herein, we review literature pertaining to the reactivity, production and prevention of these cytotoxic lipid peroxidation products in the male germline. 

## 2. Aldehydes in the Male Germline

**Figure 1:**
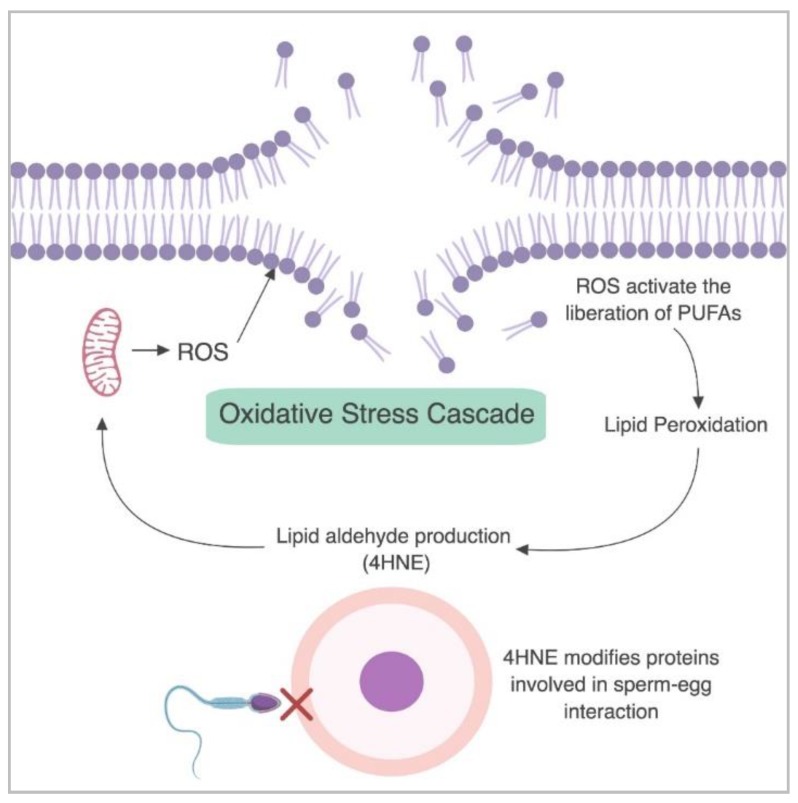
The cascade of oxidative stress in human spermatozoa. Mitochondrial reactive oxygen species (ROS) are produced and initiate the breakdown of the lipid plasma membrane. This promotes lipid peroxidation and the production of cytotoxic lipid aldehydes such as 4-hydroxynonenal (4HNE). In turn, 4HNE upregulates ROS production while causing an overall decline in cell function, ultimately impairing sperm-egg interaction. Figure created with BioRender.

In developing male germ cells and mature spermatozoa, two of the primary aldehyde products of lipid peroxidation that have been reported to cause cellular damage are MDA and 4HNE [19,22]. Increased levels of MDA are linked to a reduction in sperm concentration, normal morphology and motility [23,24]. Similarly, MDA is present at higher levels within the sperm of infertile men and is thought to initiate a loss of motility, reduction in sperm concentration and atypical morphology [24]. The levels of 4HNE within spermatozoa are positively correlated with mitochondrial superoxide formation [10], suggesting that elevated levels of 4HNE place sperm cells under increased levels of oxidative stress. Accordingly, the presence of 4HNE has been linked to numerous adverse effects on sperm function including a decline in motility, morphology, the capacity to acrosome react, and to engage in interactions with the zona pellucida of oocytes [19,25,26]. Specifically, the exposure of biomolecules to 4HNE stimulates an upregulation of mitochondrial ROS, generating a cascade of oxidative stress within human spermatozoa [19], as depicted in Figure 1.

Overproduction of 4HNE within sperm cells is linked to a reduction in sperm motility [26] and sperm-zona pellucida (ZP) interaction mediated by the molecular chaperone heat shock protein A2 (HSPA2) [25], and an increase in cell death [19]. There are several non-enzymatic pathways for aldehyde production, the best characterized being Fenton reactions, whereby ferrous iron (${\mathrm{Fe}}^{2+}$) within the cell is able to interact with lipids (LOOH) allowing the formation of lipid hydroperoxides (LO^•^) as shown in Equation (1) [27] and the production of aldehydes (as reviewed by Spiteller and Ayala et al.) [20,27].

(1) $$\mathrm{LOOH}+{\mathrm{Fe}}^{2+}\to \mathrm{LO}•\;+{\mathrm{Fe}}^{3+}+\mathrm{OH}•$$

Importantly, 4HNE is also produced via enzymatic pathways involving lipoxygenases such as arachidonate 15-lipoxygenase (ALOX15), with several studies highlighting that key metabolites such as 13-HpODE lead to the production of 4HNE [20,28], while MDA appears to be synthesized independent of lipoxygenase activity [29]. 4-hydroxynonenal is considered to be the most toxic lipid aldehyde produced within the cell [30]. This is due, at least in part, to its reactivity and subsequent capacity to alkylate proteins, generate DNA damage and ultimately cause cell death [19,25,26,31]. The reactivity of 4HNE lies in its ability to form Schiff bases and/or participate in Michael reactions. The preferential biological targets for these reactions are proteins, specifically primary amines such as lysine, but reactions with cysteine and histidine amino acid residues are also common [32,33]. A particular target for 4HNE adduction is succinate dehydrogenase (SDH) [19], a key protein in the electron transport chain within the mitochondria. Excess 4HNE has been shown to form adducts with SDH, which result in a loss of function. This ultimately facilitates electron leakage to electron acceptors in an unregulated fashion, increasing the production of ROS and eventually precipitating a state of oxidative stress within the cell [19]. Another such example in human spermatozoa is the molecular chaperone HSPA2 [34], which is also targeted for adduction by 4HNE [25]. Such modifications of HSPA2 results in a loss of its chaperoning ability and thus significantly attenuates the ability of the protein to coordinate the expression of receptors on the sperm surface; a maturational event that is critical for sperm-egg recognition [25]. Ultimately, this sequence of events culminates in a severely reduced capacity for fertilization [25,26].

Overall, the production of 4HNE has been shown to have a direct effect on the function of its protein targets, leading to cellular damage in the male germline as well as other cell types. Therefore, targeting the lipoxygenases responsible for the production of these reactive aldehydes may be an important strategy to both counter the onset of oxidative stress and reduce the cellular damage generated by 4HNE. Here, we investigate in more detail the involvement of lipoxygenase proteins in the enzymatic production of 4HNE.

## 3. Mechanisms for the Generation of 4HNE: A Focus on Lipoxygenase Proteins

Lipoxygenase proteins are a highly conserved family of enzymes that are ubiquitously found in plants [35,36], fungi [37] and mammals [38], but are rarely found in lower eukaryotes and prokaryotes and are absent in archaea and viruses [38,39,40]. Mammalian lipoxygenases typically consist of singular polypeptide chains, two functional domains and a molecular mass of ~75–80 kDa [41,42,43]. The C terminus contains the catalytic domain, while the N terminus is involved in processes governing membrane binding and interaction with substrates [42]. The catalytic pocket of the enzyme coordinates a single, non-heme containing iron atom per molecule [41,44], which is actively involved in the redox reactions necessary to facilitate the selective peroxidation of PUFAs [41,45]. However, this two domain structure is not conserved across all prokaryotes [46], and the presence of manganese replaces iron in the catalytic site of some fungal lipoxygenases [47,48,49]. The classification system of lipoxygenases (ALOX-n) defines the carbon position where oxygenation takes place along the PUFA chain. Table 1 indicates the known paralogs of human lipoxygenases, their substrates and metabolic products.

PUFA substrates for ALOX15 include ω-6 fatty acids such as arachidonic and linoleic acid and the ω-3 fatty acid, docosahexaenoic acid [50]. The mechanisms underpinning lipoxygenase function are still not entirely understood. However, it is clear that the iron center can alternate between ferric (Fe^3+^/active) and ferrous (Fe^2+^/inactive) forms [43] and this redox activity assists in hydrogen abstraction (L-H→L) of PUFAs when the iron atom undergoes a reduction (Fe^3+^→Fe^2+^) [41,51]. This reaction mechanism anticipates that the enzyme is converted back to its active form through oxidation of the iron center (Fe^2^^+^→Fe^3+^) and oxygenation (L→LOO) of the PUFA [41,43]. Importantly, recent studies assessing the enzymatic action of ALOX15 have identified binding sites for allosteric inhibition, which will allow for further insight into its specific activity [52,53].

Numerous studies have focused on the possible pathogenic implications of the lipoxygenase family, with a key focus on ALOX5 due to its role in the biosynthesis of leukotrienes, which are inflammatory mediators [61]. Leukotrienes can cause pathological inflammatory responses in diseases such as cystic fibrosis [62], inflammatory bowel disease [63] and asthma [64], thereby presenting a relationship between lipoxygenase activity and immune responses. Chronic inflammation has the potential to place cells under stress, which in turn can promote cell death or abnormal cell differentiation [65]; the latter of these, in turn, has the potential to promote tumorigenesis [66]. In the case of ALOX15, several studies have implicated this protein in the inflammation pathway of diseases such as colorectal cancer [67], prostate cancer [68] and chronic myeloid leukemia [69]. However, while the formation of 14,15-leukotrienes from ALOX15 has been proposed [70], the biological relevance of these specific compounds has not yet been explored. 

Interestingly, ALOX15 activity has also been linked to obesity as the enzyme is highly expressed in omental tissue compared to the subcutaneous fat layer of obese patients [71]. Accordingly, analysis of ALOX15 transgenic mice supports a link between inflammation, obesity and insulin resistance [72]. Indeed, this study proposes that an overexpression of ALOX15 stimulates the production of pro-inflammatory mediators, which promote insulin resistance induced through a high fat diet [72]. In turn, insulin resistance results in an overall increased risk in developing type 2 diabetes and obesity [72]. It is now well established that obesity can have detrimental impacts on both maternal and paternal fertility, as well as embryo health and development [73,74]. Obesity in males, is linked with an increased time to conception and a decrease in sperm function [73]. With these lines of evidence, the activity of ALOX15 may have a systemic and indirect effect on male infertility through obesity, alongside the direct effects it may have within the male germline through 4HNE production. The imperative for understanding mechanisms of male infertility is further supported by the growing evidence that male fertility status may in fact be an effective indicator of general health of the individual [75,76,77]. Specifically, studies assessing the fertility of more than 40,000 males have revealed that important semen parameters such as volume, cell count, and morphology are directly correlated with life expectancy [76]. A similar link has also been observed in the context of the prevalence of infertility in diseased men experiencing inflammatory bowel disease [78], obesity [79,80,81], diabetes [82], hypertension and also sexually transmitted diseases such as chlamydia [83], human immunodeficiency virus (HIV), and hepatitis C [84]. Such data suggest that drivers of poor fertility may originate in systemic issues rather than being restricted to the male reproductive tract, again emphasizing the importance of gaining a better understanding of the fundamental aspects of infertility and its origins.

At this time, literature on ALOX15 in the male germline is very scarce. Nevertheless, analysis of ALOX15 within mature spermatozoa has indicated a putative role for the enzyme within the cytoplasmic droplet of mammalian species such as boar [85] and mouse [86]. These studies suggest that ALOX15 works in concert with the ubiquitin pathway to cause organelle degradation, assisting in the removal of the cytoplasmic droplet [85]. Additionally, the production of an ALOX15 knockout mouse model has shown that the loss of this enzyme does not compromise sperm production per se. However, the spermatozoa produced from null males exhibited atypical cytoplasmic droplet degradation during epididymal transit [85]. Earlier work provided an indication that the bull sperm acrosome reaction may be suppressed following lipoxygenase inhibition [87]. However, these data must be interpreted with caution owing to the use of non-specific lipoxygenase inhibitors, and the absence of substantiating evidence to illuminate the direct role of ALOX15 in the induction of acrosomal exocytosis. Recent studies have suggested a possible link between this lipoxygenase enzyme and oxidative stress propagation in human spermatozoa [88] and in mouse germ cells [89,90]. Using an immortalized spermatocyte cell line [GC-2spd(ts)], we have demonstrated that the treatment of these cells with an ALOX15 inhibitor resulted in significant reductions in 4HNE protein modifications and subsequent oxidative stress cascades [89]. However, direct evidence of the ability of PD146176 to inhibit ALOX15 function is yet to be established and further work is required to verify the function of ALOX15 in rodent models [91]. Despite these shortcomings, using a double knockout study, Brütsch and colleagues have established a clear link between ALOX15 activity and a key antioxidant, glutathione peroxidase 4 (GPX4), in mouse germ cells [90]. In this study, the inactivation of *Gpx4* (genotype *Gpx4*^+/−^) led to significant sperm defects, including marked reductions in sperm motility (total, rapid and progressive). These *Gpx4*^+/−^ mice correspondingly exhibited significantly reduced litter sizes compared to wild type mice. However, both the motility attributes and the litter sizes of the animals were significantly improved following a simultaneous knockout of the *Alox15* gene (i.e., genotype *Gpx4^+/−^/Alox15^−/−^*), thus implicating ALOX15 in the mediation of oxidative damage in the mouse [90].

In addition to these animal studies, we have recently reported on a possible role for ALOX15 in human spermatozoa using the selective ALOX15 inhibitor 6,11-dihydro[1]benzothiopyrano[4,3-b]indole (PD146176, Tocris). This (PD146176) inhibits ALOX15 through non-competitive and non-antioxidant means [92,93] and has previously been shown to reduce the production of specific ALOX15 metabolites such as 15-HPETE [94] and 13-HODE [95]. Though minimal studies have used PD146176 in spermatozoa, the use of this inhibitor in conjunction with an oxidative challenge has been documented to give rise to significant ROS reductions in neuronal cells [96]. This is consistent with our findings in human spermatozoa, that under oxidative stress conditions ALOX15 inhibition significantly decreased ROS production and lipid peroxidation levels while also improving the functional competence of sperm populations including their motility, acrosome reaction rates and ability to undergo sperm-egg interaction processes [88]. Importantly, such studies are also consistent with those completed in the context of neurological disorders such as Alzheimer’s disease, where disease progression often relies on oxidative stress and the production of 4HNE. This lipid peroxide end product has been shown to promote the production of amyloid beta plaques and neuronal death [97,98,99]. Strikingly, these studies have demonstrated reduced amyloid plaque production with significant improvements in memory deficits through the inclusion of the same ALOX15 inhibitor, PD146176 [100,101]. These data provide further evidence for the use of PD146176 as a potential therapeutic means to prevent pathologies induced through oxidative stress and lipid peroxidation.

## 4. Protecting the Germline from 4HNE-Induced Damage

There are increasing numbers of couples using assisted reproductive technologies (ART) to achieve conception. This has led to more than 5 million births since the invention of this technology [102]. While ART has undoubtedly changed the lives of many, such technologies are highly expensive and have a live birth success rate of no more than ~30% [103,104]. There may also be a level of risk associated with assisted conception where numerous studies have confirmed that higher levels of DNA damage are present in men with subfertility [105]. This presents the possibility that ARTs could be inadvertently using damaged sperm cells which may elevate the risk of adverse health outcomes for the offspring conceived through assisted reproduction [106,107]. Additionally, the lack of selection pressure on the gametes may eventually propagate further fertility issues for future generations. A major origin of sperm cell damage arises through the onset of oxidative stress. Congruent with DNA fragmentation, markers of oxidative stress are also elevated in the infertile population [108]. It is therefore without surprise that antioxidant supplementation is an extensively studied area for the mitigation of male infertility. Table 2 summarizes numerous studies that have examined male fertility following antioxidant supplementation and their corresponding reproductive outcome. This table was collated through examination of external literature as well as analysis of a variety of detailed reviews [109,110,111]. Interestingly, only 5 out of the 28 investigated studies presented improvements to pregnancy and live birth rates following antioxidant supplementation, with positive effects associated with astaxanthin [112], l-carnitine + l-acetyl carnitine [113], Menevit^®^ [114], vitamin E [115] and zinc sulphate [116]. While some studies observed very high levels of variability when measuring semen parameters, the studies focusing on l-glutathione, lycopene, N-acetylcysteine + selenium, ubiquinone, selenium and zinc sulphate, consistently presented improvements in at least one or more semen parameter [116,117,118,119,120,121,122]. Other studies using antioxidants such as Co-enzyme Q10, folic acid + zinc sulphate, lycopene and l-carnitine + l-acetyl carnitine showed variation in effects between trials, with some studies reporting improvements to semen parameters [113,118,123,124,125,126,127,128], while others showing no positive effects [129,130,131]. Some of this variation may be attributed to intrinsic variations within each trial, such as dose regimes, methodology and the duration of treatments. Nonetheless, this variability, combined with a lack of clinical success in terms of increased pregnancy rates and live birth rates, highlights a clear need for further investigation into effective alternative strategies to prevent, or at least limit, ROS production in the male germline to improve a large subset of male fertility issues.

In seeking to account for the lack of consistent clinical success using regimens based on single antioxidant supplementation as a means for combating male infertility, it is possible that the scavenging nature of antioxidants [132] fails to provide direct protection against the cascades of lipid peroxidation and 4HNE production that ensue under conditions of oxidative stress. Interestingly, nucleophiles such as penicillamine have been shown to successfully reduce cellular ROS in both human spermatozoa and in oocytes [19,25,133,134]; effects that manifest in the recovery of sperm-oocyte interaction in vitro [25]. However, this antioxidant has serious off-target toxicity concerns [135], and thus investigation into the clinical utility of penicillamine is not possible. Another novel antioxidant formulation therapy in the male germline is Fertilix^®^ (Cell Oxess, Ewing, USA), which has been shown to protect against DNA damage in antioxidant deficient mice [136]. However, clinical trials have yet to be performed to establish whether this therapy is an appropriate method for treating infertile men. Among alternative methods that have shown promise in protecting somatic cells from diseases linked to lipid peroxidation-dependent mechanisms [137,138,139], is the stabilization of the lipid membrane through deuteration [140]. Such success provides an important precedent to investigate the efficacy of this strategy to protect sperm membranes.

## 5. Conclusions

**Figure 2:**
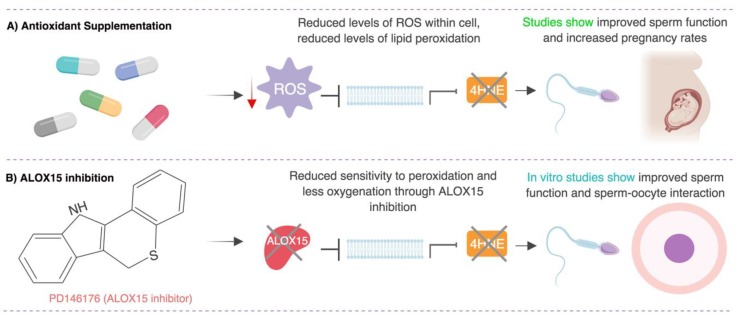
Potential strategies to protect human spermatozoa against oxidative stress. This model explores two strategies to protect against oxidative stress: antioxidant supplementation and arachidonate 15-lipoxygenase (ALOX15) inhibition. (**A**) Antioxidant supplementation has been shown to reduce levels of ROS, hence lipid peroxidation may be prevented, or its products scavenged, allowing sperm function to be improved. (**B**) ALOX15 inhibition in human sperm has been demonstrated to reduce lipid peroxidation and improve sperm function and sperm-oocyte interaction in vitro. Figure created with BioRender.

In this review we discuss strategies to alleviate oxidative stress in males suffering from fertility issues (summarized in Figure 2). Here we provide new perspectives on the lipoxygenase–lipid peroxidation pathway and discuss the merit of ALOX15 as a potential therapeutic target that could be exploited to protect human spermatozoa against oxidative stress, a key origin of poor cell function. Overall, this review highlights the importance of correct lipid metabolism in the maintenance of sperm function and fertility and provides the impetus to explore targeted, lipid-based antioxidant approaches to prevent lipid-peroxidation induced changes in the male germline. 

## Figures and Tables

**Table 1:** Paralogs and metabolites of the family of human lipoxygenase enzymes.

Lipoxygenase Enzyme ^1^	Substrates ^2^	Metabolic Products	References
ALOX5	AALA EPA	5-HpETE, 5-HETE and DGLA, Leukotrienes	[43,54]
ALOX12	AALA EPA DGLA	12-HpETE, 12-HETE, 12-HPETre, 12-HEPE, 12-HPOTrE	[50,54,55]
ALOX15	AALA DHA	15-HpETE, 15-HETE, 13-HpODE, 13-HODE, 17-HpDHA	[50,54,56,57]
ALOX12B	AALA LωHC	12R-HpETE, 12R-HETE, 9R-HpODE, 9HωHC	[50,54,58]
ALOX15B	AA	15-HpETE, 15-HETE	[50,54,59]
ALOXE3	12(R)HpETE 9HωHC	Epoxyalchohols (metabolism of 12(R)-HpETE) 9TEHωHC	[54,60] ^3^

^1,2^ Paralogs of the lipoxygenase family are shown along with their corresponding substrates of arachidonic acid (AA, red), linoleic acid (LA, green), eicosapentanoic acid (EPA) and docosahexaenoic acid (DHA). Abbreviations: arachidonate lipoxygenase (ALOX), epidermal type lipoxygenase (ALOXE), hydroperoxyeicosatetraenoic acid (HpETE), hydroxyeicosatetraenoic acid (HETE), 12-hydroxyeicosapentaenoic acid (HEPE), Hydroperoxyeicosatrienoic acid (HPEtrE) hydroperoxyoctadecadienoic (HpODE), hydroxyoctadecadienoic (HODE), 12-hydroperoxy-9Z,13E,15-octadecatrienoic acid (12-HPOTrE) hydroperoxydocosahexaenoic acid (HpDHA) and Dihomo-γ-linoleic acid (DGLA), Linoleyl-ω-hydroxy ceramide (LωHC), 9(R)-hydroperoxyllinoleoyl-ω-hydroxy ceramide (9HωHC), 9(R)-10(R)-trans-epoxy-11E-13(R)-hydroxylinoleoyl-ω-hydroxy ceramide (9TEHωHC). ^3^ It is noted that under normoxic conditions ALOXE3 does not exhibit lipoxygenase activity [60].

**Table 2:** Benefits of antioxidant supplementation for male fertility. A summary of tested antioxidants and their relative success for the improvement of male fertility as reviewed by Ahmadi et al., Ross et al., and Majzoub and Agarwal [109,110,111].

Antioxidant	Outcomes	References
Astaxanthin	Increased pregnancy rates	[112]
	Reduced oxidative stress	[112]
Co-enzyme Q10	Improved sperm motility	[123]
	Improved sperm concentration and morphology	[124]
	Altered antioxidant enzyme activity	[124,129]
	No improvements to sperm motility, concentration or morphology	[129]
Folic Acid + Zinc Sulphate	Improved sperm concentration	[125,126]
	No improvements to sperm motility, concentration or morphology	[130]
l-Glutathione	Improved motility	[117]
l-Carnitine + l-acetyl carnitine	Increased motility (progressive and total)	[127,128]
	No changes to motility or concentration	[131]
	Increased pregnancy rates and improved sperm concentration, motility and morphology	[113]
Lycopene	Improved sperm motility and concentration	[118]
Menevit	Improved pregnancy rates	[114]
N-acetylcysteine	Increased sperm concentration	[141]
	No significant increase in spontaneous pregnancies	[141]
	Improved sperm volume, motility and viscosity	[142]
	Reduced oxidative stress	[142]
N-acetylcysteine + Selenium	Improved sperm motility, concentration and morphology	[119]
Ubiquinone	Improved sperm motility, concentration and morphology	[143]
Vitamin E	Improved sperm motility	[115]
	Improved pregnancy rates	[115]
	Decreased lipid peroxidation products	[115]
Vitamin E + Vitamin C	No changes to motility or concentration	[144,145]
	Reduced DNA damage	[144]
	Improved ICSI outcomes	[146]
Vitamin E + Selenium	Improved morphology	[147]
	Improved sperm motility	[148]
	Decreased lipid peroxidation products	[148]
Selenium	Improved sperm motility	[120]
	No changes to sperm concentration	[120]
Zinc Sulphate	Improved semen volume, sperm motility and concentration	[116,121]
	Improved live birth rate	[116]
	Altered antioxidant enzyme activity	[122]
